# Effect of Different Scaling Methods on the Surface Topography of Different CAD/CAM Ceramic Compositions

**DOI:** 10.3390/ma16082974

**Published:** 2023-04-08

**Authors:** Passent Ellakany, Nourhan M. Aly, Maram M. Alghamdi, Shahad T. Alameer, Turki Alshehri, Sultan Akhtar, Marwa Madi

**Affiliations:** 1 Department of Substitutive Dental Sciences, College of Dentistry, Imam Abdulrahman Bin Faisal University, Dammam 32210, Saudi Arabia; 2 Department of Pediatric Dentistry and Dental Public Health, Faculty of Dentistry, Alexandria University, Alexandria 21527, Egypt; 3 College of Dentistry, Imam Abdulrahman Bin Faisal University, Dammam 32210, Saudi Arabia; 4 Department of Biophysics, Institute for Research and Medical Consultations (IRMC), Imam Abdulrahman Bin Faisal University, Dammam 31441, Saudi Arabia; 5 Department of Preventive Dental Sciences, College of Dentistry, Imam Abdulrahman Bin Faisal University, Dammam 32210, Saudi Arabia

**Keywords:** CAD/CAM, ceramics, dental scaling, ultrasonics, surface roughness

## Abstract

This study evaluated the effect of ultrasonic and manual scaling using different scaler materials on the surface topography of computer-aided designing and computer-aided manufacturing (CAD/CAM) ceramic compositions. After scaling with manual and ultrasonic scalers, the surface properties of four classes of CAD/CAM ceramic discs: lithium disilicate (IPE), leucite-reinforced (IPS), advanced lithium disilicate (CT), and zirconia-reinforced lithium silicate (CD) of 1.5 mm thickness were evaluated. Surface roughness was measured before and after treatment, and scanning electron microscopy was used to evaluate the surface topography following the performed scaling procedures. Two-way ANOVA was conducted to assess the association of the ceramic material and scaling method with the surface roughness. There was a significant difference in the surface roughness between the ceramic materials subjected to different scaling methods (*p* < 0.001). Post-hoc analyses revealed significant differences between all groups except for IPE and IPS where no significant differences were detected between them. CD showed the highest surface roughness values, while CT showed the lowest surface roughness values for the control specimens and after exposure to different scaling methods. Moreover, the specimens subjected to ultrasonic scaling displayed the highest roughness values, while the least surface roughness was noted with the plastic scaling method.

## 1. Introduction

Computer-aided designing and computer-aided manufacturing (CAD/CAM) technology became a cornerstone in the fabrication of ceramic restorations in prosthetic dentistry [1,2]. The higher strength of the resulting restorations with improved hardness, marginal fit, smoothness, esthetics, and color stability, as well as the production of restorations in a short duration and the limited number of visits, attracted dentists to the use of this digital technology [1,3,4,5].

The type of restoration and the location of the natural tooth requiring prosthesis are influential factors in selecting the optimum dental ceramic composition [6,7,8,9]. The use of ceramic materials in dental restorations has increased due to their excellent combination of esthetic, biological, and mechanical properties, such as wear resistance and stiffness, making them an important material for rehabilitation in the mouth [10]. Lithium disilicate and leucite-reinforced CAD/CAM ceramics have shown favorable results when used in the fabrication of esthetic restorations of moderate strength in the anterior region extending to the maxillary premolars and the short span 3-unit fixed partial dentures (FPDs) [7,8,9,11]. These ceramics were developed in different shades and multiple translucencies in order to match the esthetic appearance and brightness of anterior teeth [7,12]. The evolution of zirconia-reinforced lithium silicate ceramics incorporating zirconia fillers in the lithium silicate ceramic matrix enhanced the flexural strength of the resultant ceramics while maintaining their high translucency and esthetic features [7,13,14,15].

Recently, a new glass-ceramic material named advanced lithium disilicate glass-ceramic (CT; CEREC Tessera) was introduced to the dental market [16,17,18]. This block is composed of new crystal particles called virgilites which are added to the lithium disilicate crystals forming lithium aluminum silicate ceramics [16,17,18,19]. The high flexural strength, translucency matching the tooth-like appearance, and fast processing duration were the unique properties of this ceramic material, as reported by the manufacturer [17,19].

The time efficiency, predictability, and economically attractive treatment options are important aspects in CAD/CAM dentistry. CEREC’s newly introduced advanced lithium disilicate ceramic produces an esthetically pleasing and clinically excellent restoration when used in conjunction with the chairside economical restoration of esthetic ceramics (Chairside EREC)/ceramic reconstruction (CEREC) system [20].

Besides having a high flexural strength, advanced lithium disilicate ceramic is reinforced with virgilite crystals in order to satisfy the esthetic demands of patients. A biaxial strength of more than 700 MPa is claimed by the manufacturer, along with improved optical properties with a remarkable sintering speed of approximately four minutes, which provides a shorter processing time and high flexural strength [20], thus, optimizing the chairside workflow. It is anticipated that new treatment options will be available for these advanced lithium disilicate ceramics.

The longevity of dental restorations is one of the most important criteria in evaluating the success of the definitive restorations. Achieving this criterion depends mainly on the patient’s oral hygiene together with the mechanical properties of the materials used in fabricating the definitive prosthetic restorations [21]. Routine scaling and root planing are critical in preserving the normal periodontal health through the removal of the biofilm and calculus deposits, in addition to pocket debridement [22]. These methods can be manually performed using hand metallic curettes or piezoelectric ultrasonic scalers and different types of scaling tips such as plastic, stainless steel, or titanium tips [23,24]. All of these scalers might compromise the surface roughness of the dental ceramic restorations and lead to unfavorable consequences [25,26]. Additionally, the material of the scaling tips has specific indications; for example, plastic tips are used particularly for implant-supported restorations to avoid any harmful consequences on the longevity and osseointegration of dental implants [27].

The roughened surface of dental ceramics can lead to plaque accumulation and bacterial colonization, compromising the biocompatibility of the restoration, color stability, and esthetics [28,29]. Moreover, these irregularities may incorporate microcracks, leading to fracture and failure of the restorations under masticatory loads [30].

The aim of this study was to evaluate the effect of different scaling methods and scaler materials on the surface topography of four CAD/CAM ceramics. The first null hypothesis states that there would be no significant difference in the surface roughness of CAD/CAM dental ceramics after exposure to different scaling tip materials. Secondly, the surface roughness would not show significant differences among the different dental ceramic compositions following the use of various scaling methods.

## 2. Materials and Methods

**Figure 1:**
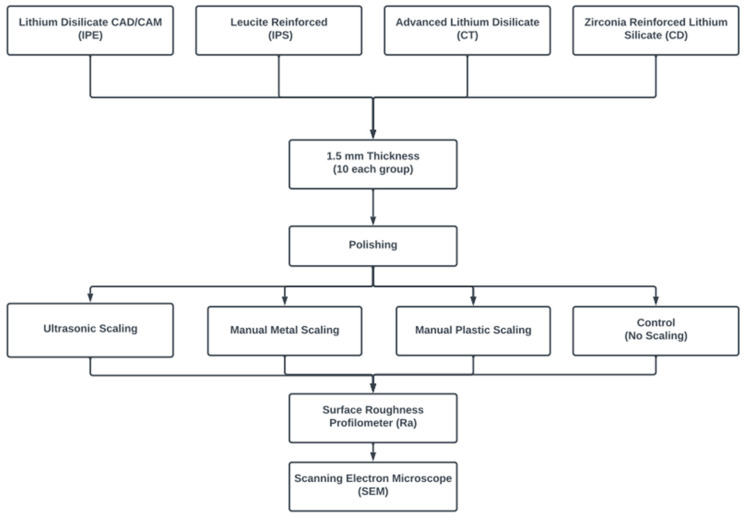
Study design flowchart.

The current study evaluated the surface properties of four classes of CAD/CAM ceramics following different scaling methods of variable scaling tip materials. Lithium disilicate (IPE; IPS Emax CAD, Ivoclar Vivadent, Schaan, Liechtenstein), leucite-reinforced (IPS; IPS Empress CAD, Ivoclar Vivadent, Schaan, Liechtenstein), advanced lithium disilicate (CT; CEREC Tessera, Sirona Dentsply, Milford, DE, USA), and zirconia-reinforced lithium silicate (CD; Celtra Duo, Sirona Dentsply, Milford, DE, USA) blocks of shade A1 and low translucency were sectioned into 1.5 mm thicknesses (Figure 1). The sample size was calculated assuming a 5% alpha error and 80% study power. Ellakany et al. [31] reported mean (SD) surface roughness = 0.67 (0.05), 0.71 (0.07), 0.56 (0.06), and 0.59 (0.04) for IPE, CD, CT, and IPS, respectively. Based on a comparison of the means, using a 2-tailed test, the minimum sample size was calculated to be 4 per group but was increased to 5 to make up for laboratory processing errors. The total sample size required = number per group × number of groups × number of scaling methods = 5 × 4 × 4 = 80 samples. The sample size was calculated using G*Power (Version 3.1.9.7, Heinrich-Heine-Universität Düsseldorf, Düsseldorf, Germany) [32].

### 2.1. Sectioning of Samples

The ceramic blocks were sliced using the precision cutting machine (Isomet 5000, Buehler, Lake Bluff, IL, USA) under proper water coolant. The resultant samples were 1.5-mm thick with the exact measurements of the machined block according to the International Organization of Standards (ISO standards 6872:2015) [33]. Polishing of the samples was performed for both sides using 500 grits of silicon carbide discs for 1 minute at a speed of 200 rpm under a water coolant system using the polishing machine (MetaServ 250 Grinder-Polisher with Vector Power Head, Buehler, IL, USA). All samples were inserted in a ceramic furnace (Programat EP5010, Ivoclar Vivadent) to reach the full crystallization stage according to the manufacturer’s guidelines. The second polishing procedure was applied for the same two sides using carbide discs of various grits (400 and 600 grits) in a moist environment for 1 minute at 200 rpm speed using the same polishing machine stated above. All samples were measured with a digital caliper (Mitutoyo Corp, Kawasaki, Japan) before applying any test to ensure the accuracy of the samples’ dimensions and that they were within 0.05-mm thick.

### 2.2. Surface Roughness Measurements (Ra)

Initial surface roughness readings of the ceramic samples were detected using a non-contact optical profilometer (Contour Gt-K 3D optical profiler; Bruker Nano GmbH, Berlin, Germany). The Ra of each sample was performed by measuring three readings at different locations, where the speed was 0.5 mm/s and the cutoff was adjusted at 0.8 mm [31,34]. Then, the average of the three readings was calculated.

### 2.3. Scaling Procedure

The samples were divided into four subgroups from each ceramic type according to the type of dental scaler used; metal stainless steel tip (H3/H4 Jacquette Scaler, Hu-Friedy Mfg. Co., Chicago, IL, USA), plastic tip made of Plasteel which is a high-grade, unfilled resin material (Implacare II LG1/2, Hu Friedy Mfg. Co., Chicago, IL, USA), piezoelectric unit (Piezo Scaler Tip 201, KaVo PiezoLED Ultraschall Scaler, Kaltenbach & Voigt GmbH, Biberach, Germany), and the last subgroup was the control group where samples were not subjected to the scaling procedure. Thus, each ceramic type would include 5 samples in each subgroup, and, as both surfaces were scaled, the total would be 10 samples in each group. All scaling procedures were performed by a single trained operator to standardize the scaling protocol. Manual scaling was applied on both sides of the samples using pull strokes with 75° working angulation and moderate lateral pressure for 40 strokes (approximately 40 s) [26,35,36]. The samples were scaled with the ultrasonic tip using water irrigation where tips were used at a 0° angle [37,38,39] with moderate power of standard lateral force approximately 0.2 N [29] and the same number of 40 strokes as the manual scaling performed. Scaling was carried out from the center of the sample to the borders [39]. The curettes and piezoelectric tips were replaced with new ones after five times of use. After scaling was performed, the samples were cleaned under tap water for five minutes followed by ultrasonic cleaning for ten minutes [29]. The surface roughness of all samples on both sides was measured by the same protocol mentioned for each scaling method.

### 2.4. Scanning Electron Microscopy (SEM)

The effect of different scaling tips on the surface topography of different CAD/CAM ceramic specimens was evaluated using scanning electron microscopy (SEM model; Inspect S50, FEI Company, Moravia, Czech Republic) at accelerating voltage 30 KV. The specimens were gold-coated before their examination in order to minimize the charging effect and to improve the image quality.

### 2.5. Statistical Analysis

Normality was checked for all variables using descriptive statistics and plots (histogram, boxplots, and Q-Q plots). All variables showed normal distributions, so the mean and standard deviation (SD) were calculated and parametric analysis was adopted. Comparisons of the surface roughness between the four studied ceramics were conducted using one-way ANOVA, followed by multiple pairwise comparisons using Bonferroni adjusted significance level. Two-way ANOVA was performed to assess the association of the ceramic composition and the scaling method with surface roughness. Adjusted means, standard errors (SEs), 95% confidence intervals (CIs), and adjusted R^2^ were calculated. Significance was inferred at *p*-value < 0.05. Data were analyzed using IBM SPSS for Windows (Version 26.0, Chicago, IL, USA)).

## 3. Results

**Figure 2:**
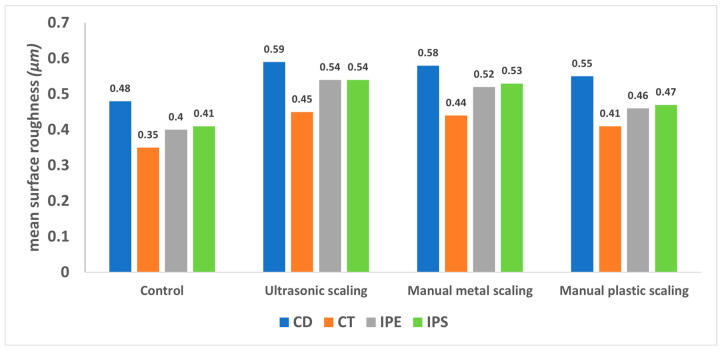
Surface roughness of the studied ceramic compositions. CD; zirconia-reinforced lithium silicate (Celtra Duo). CT; advanced lithium disilicate (CEREC Tessera). IPE; lithium disilicate (IPS Emax CAD). IPS; leucite-reinforced (IPS Empress CAD).

**Figure 3:**
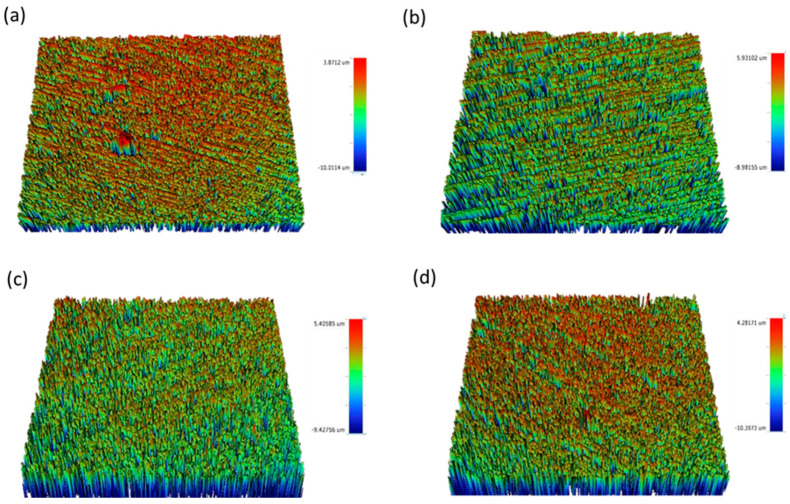
Surface roughness (Ra) of control group of each: (**a**) CD, (**b**) CT, (**c**) IPE, and (**d**) IPS. CD; zirconia-reinforced lithium silicate (Celtra Duo). CT; advanced lithium disilicate (CEREC Tessera). IPE; lithium disilicate (IPS Emax CAD). IPS; leucite-reinforced (IPS Empress CAD).

**Figure 4:**
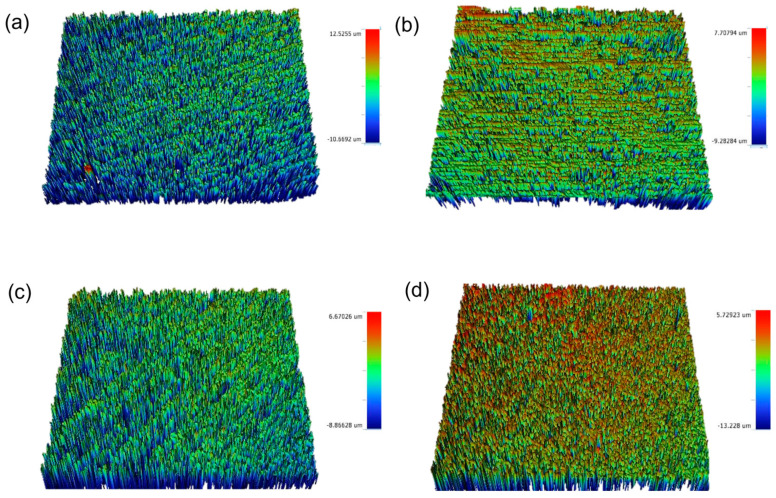
Surface roughness (Ra) of ultrasonic scaling of each: (**a**) CD, (**b**) CT, (**c**) IPE, and (**d**) IPS. CD; zirconia-reinforced lithium silicate (Celtra Duo). CT; advanced lithium disilicate (CEREC Tessera). IPE; lithium disilicate (IPS Emax CAD). IPS; leucite-reinforced (IPS Empress CAD).

**Figure 5:**
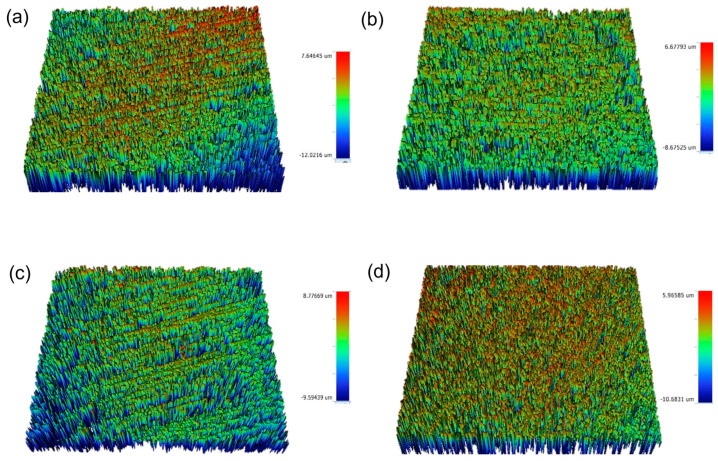
Surface roughness (Ra) of manual metal scaling of each: (**a**) CD, (**b**) CT, (**c**) IPE, and (**d**) IPS. CD; zirconia-reinforced lithium silicate (Celtra Duo). CT; advanced lithium disilicate (CEREC Tessera). IPE; lithium disilicate (IPS Emax CAD). IPS; leucite-reinforced (IPS Empress CAD).

**Figure 6:**
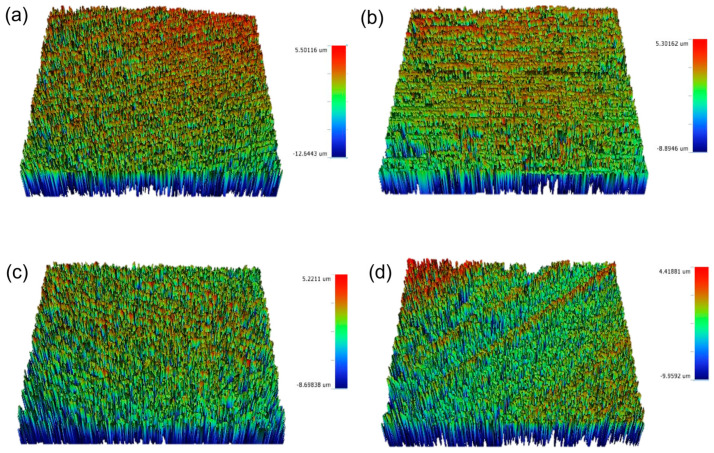
Surface roughness (Ra) of manual plastic scaling of each: (**a**) CD, (**b**) CT, (**c**) IPE, and (**d**) IPS. CD; zirconia-reinforced lithium silicate (Celtra Duo). CT; advanced lithium disilicate (CEREC Tessera). IPE; lithium disilicate (IPS Emax CAD). IPS; leucite-reinforced (IPS Empress CAD).

Figure 2, Figure 3, Figure 4, Figure 5 and Figure 6 show that there were significant differences in the surface roughness between the studied ceramics using different scaling methods (*p* < 0.001). Post-hoc comparisons revealed significant differences between all groups, except for IPE and IPS where no significant differences were detected between both groups. For the control and after exposure to different scaling methods, CD showed the highest surface roughness values, while CT showed the lowest values. Moreover, specimens exposed to ultrasonic scaling showed the highest roughness values, while the control specimens showed the lowest values.

Table 1 represents the association of the ceramic composition and scaling method with surface roughness. Regarding the ceramic materials, there was a significant difference between the studied ceramics’ compositions (*p* < 0.001). CD showed a significantly higher surface roughness than the other groups (adjusted mean = 0.55), while CT showed the lowest roughness values (adjusted mean = 0.41). No significant difference was detected between IPE and IPS (adjusted means = 0.48 and 0.49, respectively). As for the scaling methods, there were significant differences in the surface roughness between the different methods (*p* < 0.001). The highest roughness values were reported after ultrasonic scaling, followed by manual metal and plastic scalers (adjusted means = 0.53, 0.52, and 0.47, respectively), while the lowest values were reported for samples not exposed to any scaling method (adjusted mean = 0.41).

**Figure 7:**
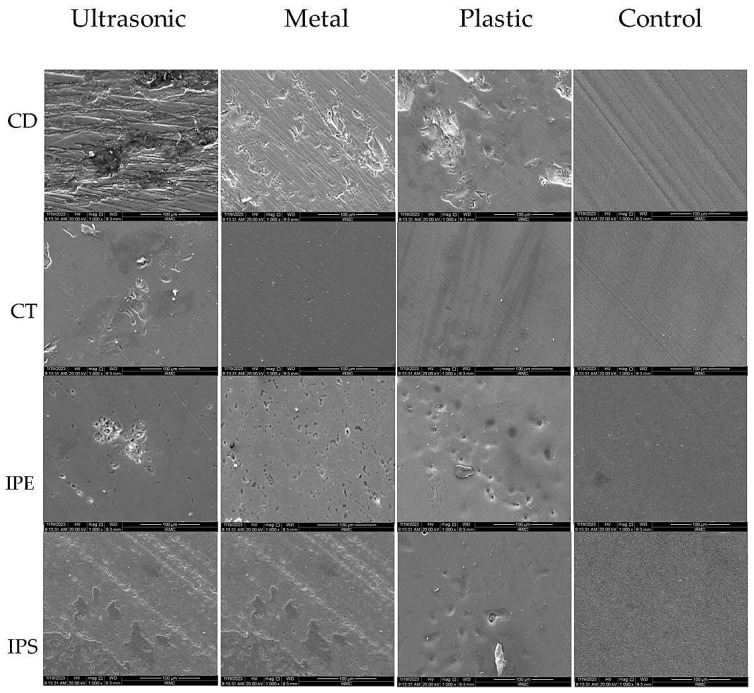
SEM images of the CAD/CAM ceramic materials (CD, CT, IPE, and IPS) treated with three treatment methods: ultrasound, manual metal, and plastic tips in addition to no scaling (control) in ×1000 magnification and scale bar 100 µm. CD; zirconia-reinforced lithium silicate (Celtra Duo). CT; advanced lithium disilicate (CEREC Tessera). IPE; lithium disilicate (IPS Emax CAD). IPS; leucite-reinforced (IPS Empress CAD).

Figure 7 shows the SEM images of the CAD/CAM ceramic materials (CD, CT, IPE, and IPS) treated with three treatment methods (ultrasound, manual metal, manual plastic tips, and control (no scaling)) displayed in magnification x1000 and a scale of 10 µm. SEM images showed significant changes in the surface structure of the samples when treated with different scaling methods. It was observed that the ultrasonic scaling produced deeper grooves and pits on the ceramic surface as compared to both manual metal and plastic tips. However, plastic tips exhibited the least changes among all ceramic types. CT was the least ceramic composition, showing surface changes by the three scaling methods when compared to the other three ceramic types (CD, IPE, IPS).

## 4. Discussion

The present study was conducted to evaluate the effect of different scaling techniques using various scaling tip materials on the surface topography of four different CAD/CAM ceramic compositions (IPE, IPS, CD, and CT). This study would help dentists in selecting the most suitable scaling instrument and technique to avoid any drastic effects that might compromise the surface smoothness of the definitive restorations. The findings showed significant differences in the surface roughness between all ceramic groups, except for IPE and IPS where no significant differences were detected between them. CD showed the highest surface roughness values, while CT showed the lowest ones. The different scaling methods caused a significant difference in the surface roughness of the tested ceramics. The samples exposed to ultrasonic scaling showed the highest roughness values followed by manual metal and plastic scalers, while the control specimens showed the lowest values. Based on these findings, both null hypotheses were rejected.

The use of ultrasonic scaling was simulating professional surface debridement, which is one of the most common supportive treatments after the final cementation of the prosthetic restorations [40]. The highest surface roughness encountered after using ultrasonic scaling in this study was in agreement with other studies [24,25,29,38,41]. The variation on the surface texture of ceramics generated by the used scalers might be related to the difference in the manufacturing material of the scalers used (plastic vs. steel) and the scaling force exerted. However, other studies were contradictory to the current results [25,42]. Zirconia exhibited the lowest surface roughness following the scaling procedure when compared to the lithium disilicate ceramics [25]. In Seol et al. [42], a low surface deterioration was noticed in scaling zirconia, porcelain samples, and other metal alloys such as gold and titanium with ultrasonic scaling. This can be attributed to the difference in the scaling duration besides the type of tested ceramics, the method of fabrication, and the polishing of the samples. Furthermore, Al Ankily et al. [43] stated that manual scaling with both titanium and stainless steel tips caused more surface roughness on natural enamel than ultrasonic scaling with the same tips. The effect of scaling is variable on the treated surfaces as enamel and dentin compositions are different from the microstructure of dental ceramics [43,44]. Titanium had a more drastic effect on the surface texture than stainless steel [43,45].

In the current study, SEM images showed that the ultrasonic tips produced deeper grooves and pits on the ceramic surface while the shallowest indentations were reposted with plastic tips; this might be due to the 0.2 N mechanical forces generated by the ultrasonic tips during vibration in linear motion resulting in pulling out of grains from the ceramic matrix, thus increasing the surface roughness.

The plastic tips caused a lower surface roughness on all tested dental ceramics in comparison to metal and ultrasonic tips. Similarly, Nakazawa et al. [29] stated that scaling with plastic tips on zirconia specimens resulted in smoother surfaces than the metallic tips. In assessing the effect of scaling on the ceramic compositions, CT had the least surface roughness after the scaling procedures. Limited studies which assessed the surface roughness of CT showed an improved wear resistance when compared to the IPE, IPS, and CD samples [16,31]. However, the surface roughness of CT in response to scaling techniques was not previously tested in the literature as CT is a recent ceramic material and needs further assessments. Both IPS and IPE did not show any significant differences in surface roughness. This can be related to the similar microstructure which is formed of a high crystalline content engaged in a bonded matrix. Nevertheless, both materials showed a higher surface roughness than CT. Micro-cracking, the partial dislodgment of lithium disilicate crystals, and an increase in the surface roughness were noted changes among IPS and IPE after scaling procedures [10,38,46]. The results also showed that CD presented the highest surface roughness among all tested ceramics with all scaling methods. The presence of 20% zirconia particles in the composition of the CD samples might be the causative factor for this roughness [47]. Yoon et al. [38] showed evident surface roughness on the zirconia samples followed by lithium disilicate pressed ceramics and metal alloys after ultrasonic scaling.

A novel dental ceramic material (CT) was tested in the current study in comparison to other commonly used ceramic types. This provides more validity to using CT ceramic material in different applications as veneers, single crowns, FPDs, and implant-supported restorations. However, further investigations are still needed to assess other mechanical and physical properties including strength, color stability, and translucency. Furthermore, assessing the effect of different scaling methods and tips intraorally will help dentists in choosing the most suitable instrument for each ceramic composition and restoration type, which will help in increasing the longevity of the dental restorations. However, the study is limited to using dental ceramics after polishing without application of the glaze material, which can affect the clinical outcomes of ceramics. Therefore, future laboratory and clinical studies are needed to assess the color stability and bacterial colonization of the tested ceramics after scaling.

## 5. Conclusions

Ultrasonic scaling resulted in a significantly higher surface roughness on all tested samples when compared to both manual metal and plastic scaling techniques. Plastic tips produced the lowest surface roughness on all ceramics, favoring its use on all restorations especially in case of implant-supported ceramic restorations. Regarding the ceramic material, CT showed the highest smoothness followed by IPE, IPS, and CD, respectively. The new composition and incorporation of virgilite particles in CT might be the cause of its promising results in the surface texture, but further assessments are still needed to verify these conclusions.

## Figures and Tables

**Table 1:** Two-way ANOVA for the association of different factors with surface roughness.

		Adjusted Mean (SE)	95% CI	*p*-Value
Ceramic Material	CD	0.55 (0.003) ^a^	0.54, 0.56	<0.001 *
	CT	0.41 (0.003) ^b^	0.41, 0.42	
	IPE	0.48 (0.003) ^c^	0.48, 0.49	
	IPS	0.49 (0.003) ^c^	0.48, 0.49	
Scaling Method	Control (no scaling)	0.41 (0.003) ^a^	0.41, 0.42	<0.001 *
	Ultrasonic scaling	0.53 (0.003) ^b^	0.53, 0.54	
	Manual metal scaling	0.52 (0.003) ^c^	0.51, 0.52	
	Manual plastic scaling	0.47 (0.003) ^d^	0.47, 0.48	

SE: Standard Error, CI: Confidence Interval. F = 349.93, *p* < 0.001 *, Adjusted R^2^ = 0.93. * Statistically significant at *p*-value < 0.05. a–d: different letters denote statistically significant differences between groups using Bonferroni adjusted significance levels. CD; zirconia-reinforced lithium silicate (Celtra Duo). CT; advanced lithium disilicate (CEREC Tessera). IPE; lithium disilicate (IPS Emax CAD). IPS; leucite-reinforced (IPS Empress CAD).

## Data Availability

Data are available upon request from the principal author.
